# Ketogenic diet as a glycine lowering therapy in nonketotic hyperglycinemia and impact on brain glycine levels

**DOI:** 10.1186/s13023-022-02581-6

**Published:** 2022-12-05

**Authors:** Emily Shelkowitz, Russell P. Saneto, Walla Al-Hertani, Charlotte M. A. Lubout, Nicholas V. Stence, Mark S. Brown, Patrick Long, Diana Walleigh, Julie A. Nelson, Francisco E. Perez, Dennis W. W. Shaw, Emma J. Michl, Johan L. K. Van Hove

**Affiliations:** 1 Section of Clinical Genetics and Metabolism, Department of Pediatrics, University of Colorado, Education 2 South, L28-4114, East 17Th Avenue, Aurora, CO 80045 USA; 2 Division of Pediatric Neurology, Department of Neurology, Center for Integrative Brain Research, Seattle Children’s Research Institute, Seattle Children’s Hospital, Seattle, WA 98105 USA; 3 Division of Genetics and Genomics, Boston Children’s Hospital, Harvard Medical School, Boston, MA USA; 4 Section of Metabolic Diseases, Beatrix Children’s Hospital, University of Groningen, University Medical Center, Groningen, Groningen, The Netherlands; 5 Department of Radiology, University of Colorado, Aurora, CO USA; 6 Section of Child Neurology, Department of Pediatrics, University of Colorado, Aurora, CO USA; 7 Department of Radiology, Seattle Children’s Hospital, University of Washington, Seattle, WA USA

**Keywords:** Nonketotic hyperglycinemia, Benzoate, Ketogenic diet, Magnetic resonance spectroscopy, Glycine, Epilepsy

## Abstract

**Background:**

Nonketotic hyperglycinemia (NKH) is a severe neurometabolic disorder characterized by increased glycine levels. Current glycine reduction therapy uses high doses of sodium benzoate. The ketogenic diet (KD) may represent an alternative method of glycine reduction.

**Aim:**

We aimed to assess clinical and biochemical effects of two glycine reduction strategies: high dose benzoate versus KD with low dose benzoate.

**Methods:**

Six infants with NKH were first treated with high dose benzoate therapy to achieve target plasma glycine levels, and then switched to KD with low dose benzoate. They were evaluated as clinically indicated by physical examination, electroencephalogram, plasma and cerebral spinal fluid amino acid levels. Brain glycine levels were monitored by magnetic resonance spectroscopy (MRS).

**Results:**

Average plasma glycine levels were significantly lower with KD compared to benzoate monotherapy by on average 28%. Two infants underwent comparative assessments of brain glycine levels via serial MRS. A 30% reduction of brain glycine levels was observed in the basal ganglia and a 50% reduction in the white matter, which remained elevated above normal, and was equivalent between the KD and high dose benzoate therapies. CSF analysis obtained while participants remained on the KD showed a decrease in glycine, serine and threonine levels, reflecting their gluconeogenetic usage. Clinically, half the patients had seizure reduction on KD, otherwise the clinical impact was variable.

**Conclusion:**

KD is an effective glycine reduction method in NKH, and may provide a more consistent reduction in plasma glycine levels than high-dose benzoate therapy. Both high-dose benzoate therapy and KD equally reduced but did not normalize brain glycine levels even in the setting of low-normal plasma glycine.

## Introduction

Nonketotic hyperglycinemia (NKH) is a severe, autosomal recessive, neurometabolic disorder caused by deficient activity of the glycine cleavage enzyme system due to pathogenic variants in *GLDC* or *AMT* [1, 2]. The enzyme complex catalyzes the conversion of glycine to carbon dioxide and ammonia while transferring the middle one-carbon to tetrahydrofolate creating 5,10-methylenetetrahydrofolate. The glycine cleavage enzyme activity is one of the major contributors to one-carbon metabolism and the most important catabolic enzyme of glycine metabolism. Deficient activity of the glycine cleavage enzyme system results in the accumulation of glycine in the fluids and tissues of the body including in the brain [3]. Most patients have the severe form of NKH and present with neonatal developmental and epileptic encephalopathy, absent psychomotor development, spasticity, and therapy-resistant epilepsy [1, 4, 5]. In contrast, patients with attenuated NKH make developmental progress to varying degrees, and typically, have easily treatable epilepsy or no epilepsy [1, 4–6].

The pathophysiology of nonketotic hyperglycinemia is complex. In addition to end-product deficiency of 5,10-methylenetetrahydrofolate [7–9], glycine toxicity both directly [10–14] and through excess glycine simulation at the allosteric site of the NR1/NR2 NMDA receptor [15–17] is hypothesized to be a major factor.

**Fig. 1:**
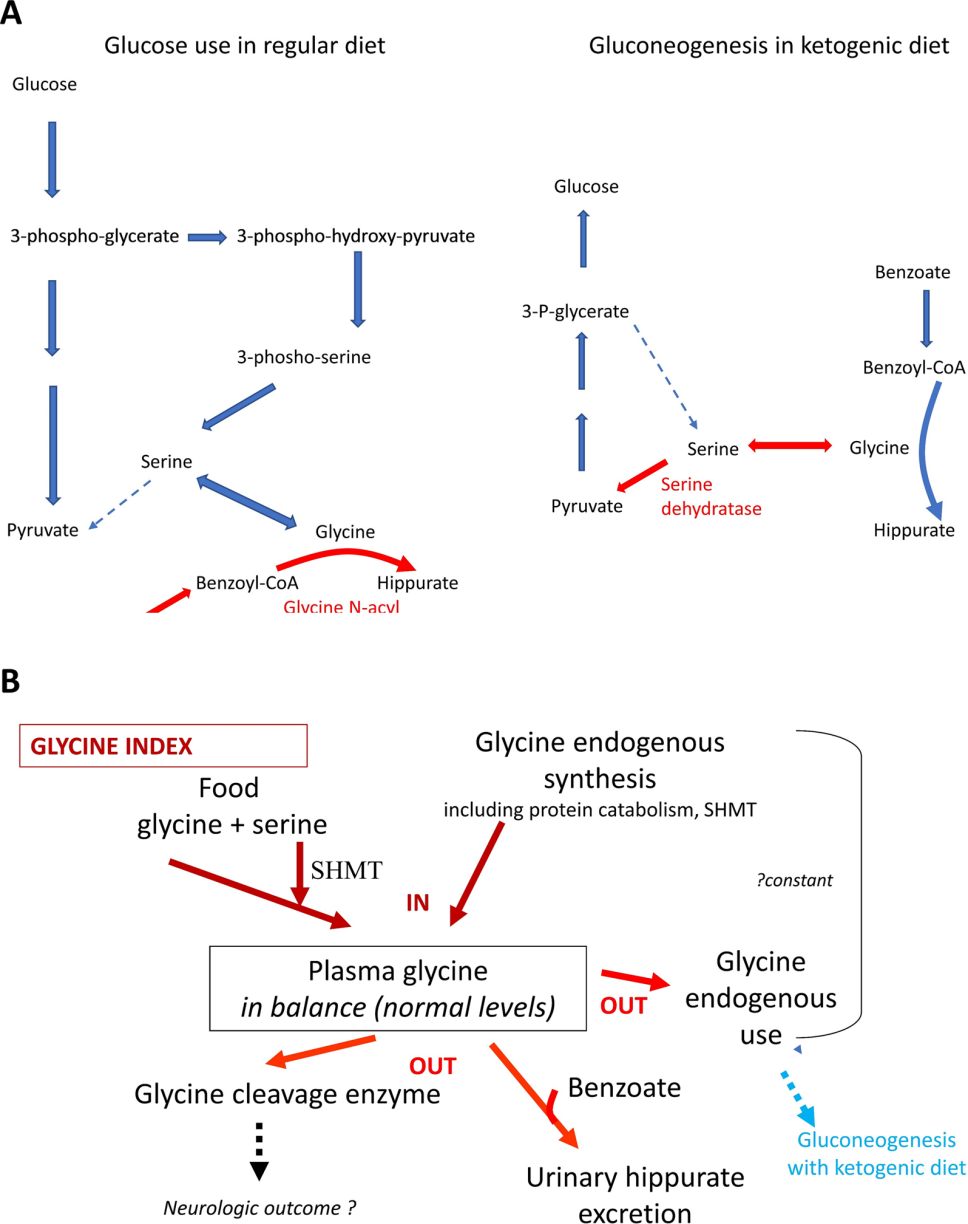
Glycine reduction strategies. **A** In patients with nonketotic hyperglycinemia, in states where glucose is present, glycine clearance is largely dependent on its conjugation to benzoyl-CoA by glycine N-acyltransferase. In states of glucose deprivation, glycine is converted to serine which is used as a starting substrate for gluconeogenesis through its conversation to pyruvate by serine dehydratase. Both glycine-N-acyltransferase and serine dehydratase are expressed primarily in the liver. **B** When glycine levels are in the target range, then the amount of glycine provided reflects the sum of dietary glycine and endogenous glycine synthesis, whereas glycine use is determined by the combination of endogenous catabolism, the residual activity of the glycine cleavage enzyme and conjugation by benzoate. This balance is reflected in the glycine index which is an individualized parameter that is determined by the weight-based sodium benzoate dose required to achieve target glycine levels relative to the amount of dietary glycine. Ordinarily this index is constant and fixed over the course of an individual patient’s lifetime. The use of the ketogenic diet increases the endogenous use of glycine for gluconeogenesis and shifts the balance of the glycine index

Current therapy centers around treating the effects of excess glycine by reducing glycine levels most commonly using benzoate and by mitigating excessive glycine-mediated neurotransmission at the NMDA-receptor using dextromethorphan or ketamine [17–22]. In mice, trans-cinnamic acid has been proposed as an alternative glycine reduction agent [23]. Glycine restricted diets have only shown marginal added benefit in lowering glycine levels, likely because dietary glycine makes only a small contribution to the total glycine pool in comparison to endogenous glycine synthesis [20] (shown in Fig. 1A). These diets also carry a risk if not carefully executed and are therefore not recommended as a method of glycine reduction [24–26]. The efficacy of glycine reduction is routinely assessed via measurement of plasma glycine levels, and the impact of therapy on the target organ, the brain, has not been well characterized.

Several case reports have described an ancillary effect of lowered plasma glycine levels with the ketogenic diet (KD), which was initiated for the treatment of refractory epilepsy in NKH patients [27–33]. We assume that this results from the use of endogenous glycine pools in gluconeogenesis (Fig. 1A) [34] A lower cerebrospinal fluid (CSF) glycine level in an NKH patient on the KD has been reported once before [30]. The impact on brain glycine levels of the KD has not yet been documented. Thus, further study of the use of the KD as a glycine reduction therapy and of its biochemical impact is needed.

It has previously been demonstrated that the KD may improve seizure control in patients with NKH, but its effect on other areas of neurodevelopment in patients with NKH has not yet been explored [27–33]. This question is of particular interest given that although a reduction of glycine levels, as traditionally accomplished via high dose benzoate therapy, leads to a decrease in seizures and improved levels of alertness and neuro-behavioral profiles, its impact on neurodevelopmental outcomes in individuals with severe NKH is minimal even when initiated neonatally [19, 20, 35–37].

Magnetic resonance spectroscopy (MRS) has been employed to detect in vivo glycine levels in brain tissue in patients with NKH in a diagnostic context [38–40]. In this study, we utilized MRS to investigate the impact of glycine reduction therapies on brain glycine levels in NKH patients. We present six children with NKH who were first treated with standard high dose benzoate therapy and then switched to ketogenic diet with low dose benzoate therapy. We review their clinical course and biochemical response in plasma, CSF and brain tissue.

## Methods

This is a retrospective observational study that included 6 individuals with NKH from four different institutions who were treated with sodium benzoate therapy and the ketogenic diet as indicated based on their clinical status between 2019 and 2021 and continuing to the present. A retrospective review of the patients’ clinical history, laboratory results and brain magnetic resonance imaging studies was performed after obtaining informed consent. Studies of US patients were done in accordance with an IRB-approved study (COMIRB 05-0790). Per institutional rule, no further ethics committee review was required for the patient at the Groningen center. Consent for publication was received from all families.

All patients were first treated with high dose benzoate aimed to achieve target plasma glycine levels between 120 and 300 µmol/L [1, 20]. Patients initiated ketogenic diet typically with a 3:1 ratio per their local clinical protocol. During KD initiation, in anticipation of a rapid decline in glycine levels, the benzoate dose was reduced to half the initial dose and then titrated based on plasma glycine levels. Plasma glycine levels were obtained at 1–2 h after a benzoate dose in order to titrate therapy to achieve target glycine levels [1, 20]. Because our primary goal was glycine reduction, we followed plasma glycine levels primarily to guide dietary or ketogenic ratio adjustments, with quantitative assessment of ketones a secondary additional outcome parameter. Clinical assessments, including lumbar puncture, magnetic resonance imaging (MRI) and MRS, and electroencephalogram (EEG) studies, were repeated based on clinical status. To study endogenous glycine use, the glycine index was calculated as described from the dose of benzoate required to achieve plasma glycine levels in the target range and the dietary intake of glycine, using the equation: (Glycine index (mmol/kg per day) = [benzoate dose (mmol) − glycine food (mmol)]/weight (kg) (Fig. 1B) [20].

Brain glycine levels were evaluated using MRS. Patients 1 and 2 underwent serial MRI with MRS. MRI and MRS studies for patient 1 (through timepoint 4) and patient 2 were performed on a 3 T Philips Ingenia MRI scanner, while MRS for patient 1 (timepoint 5) and patient 3 were performed on a 3 T Siemens Prisma Fit. Single voxel MRS with TE times of 35 ms and 144 ms was performed by placing a 1.5 × 1.5 cm voxel in two locations: medial basal ganglia and frontal white matter. The peak heights of glycine at 3.55 ppm and creatine were measured manually and ratios of glycine to creatine were calculated. Available MRS source data was post-processed using LCModel to obtain estimated metabolite concentrations [41, 42]. Control data on neonatal brain MRS glycine levels (displayed in Table 5) were extracted using identical processing from MRS data obtained over the basal ganglia of neonates imaged for possible hypoxic-ischemic injury, but who were later determined to have normal imaging and MRS findings. Data on white matter glycine levels were not available in these subjects therefore control white matter glycine levels could not be extracted. The MRS derived glycine values in the basal ganglia were considerably higher than in the white matter. As a result, the error in the white matter signal was larger, making this result technically less reliable than that of the basal ganglia. The signal at short echo time 35 ms does not separate the peaks of myoinositol and glycine, which are well separated at an intermediate echo time of 144 ms. Signals using a long echo time of 288 ms could not be used for serial glycine quantification in NKH, likely due to excessive interference over time between the 288 ms MRS signal and the evolving diffusion restriction in NKH. Unfortunately, due to a technical error, the values prior to treatment for patient 2 could not be analyzed by LCModel, and only peak height ratios are available.

## Case reports

### Case 1

The infant presented at 3 days of life (DOL 3), following an uncomplicated pregnancy and delivery, with progressive lethargy impairing her ability to maintain wakefulness and feeding. She was diagnosed with NKH based on biochemical and molecular findings (Table 1) and MRS (obtained on DOL 3) showed an elevated glycine peak (Table 5). Treatment with standard high dose sodium benzoate and dextromethorphan was initiated on DOL 5. A repeat MRS on DOL 17 showed decreased glycine levels (Table 5). Due to persistent seizures, at age 6 months brain MRI and MRS were repeated showing equivalent elevated brain glycine levels to DOL 17 (Table 5).﻿

The KD was initiated at 6.5 months. Ketosis and a robust decrease in plasma glycine levels was noted in 24 h (Table 2), and repeat CSF studies showed a decrease in glycine, serine and threonine (Table 4). Clinically, the infant’s seizures did not substantially improve, and lacosamide was added (in addition to continued use of clobazam and topiramate). While on the KD, the patient’s parents reported subjective improvements in her level of alertness and head control, and increased grasping of objects. On exam, she continued to have axial hypotonia, but her spasticity improved and her clonus resolved. At age 10 months, the patient was able to access a sugar-free formulation of dextromethorphan and higher levels of ketosis were achieved with a lower ketogenic ratio. MRI and MRS were repeated with no substantial change in brain glycine levels (Table 5). At age 13.5 months, the Bayley Scales of Infant Development demonstrated a developmental age of 1 month for gross motor and < 1 month for language and fine motor skills. The infant was able to roll in both directions and had good head control with only a minimal head lag. She had intermittent horizontal eye nystagmus but could fix and follow caregivers. She experienced multiple weeks in a row without seizures, but then would have a cluster of 4 to 5 tonic seizures in a day.

When the infant was 20 months (after 14 months on the KD), it was discontinued due to frequent vomiting and feeding intolerance experienced at higher levels of ketosis. High dose sodium benzoate therapy was resumed. A repeat lumbar puncture obtained shortly after the discontinuation of the KD as part of routine evaluations for break-through seizures showed a stable plasma and increased CSF glycine levels (Table 4). The patient remains on clobazam, lacosamide and levetiracetam. Despite the discontinuation of the KD, gastroesophageal reflux continues to be a concern. At age 27 months she is not crawling but can sit independently with mild truncal support. Pyramidal signs remain absent on exam. She has severe cortical vision impairment and babbles, but has no words. A repeat Bayley assessment showed a gross motor functional age of 6 months and fine motor of 1 month equivalent. At 2 year 9 months, she can sit independently, and grasp and hold objects.

### Case 2

Following an uncomplicated pregnancy and delivery, the infant was noted to have encephalopathy and hypotonia at birth. She developed progressive respiratory failure and apnea requiring ventilatory support by 40 h of life. MRS (obtained on DOL 5) (Table 5) and biochemical studies were consistent with a diagnosis of NKH and molecular testing revealed a missense variant in *GLDC* and an 8.8 Mb terminal deletion of 9p encompassing 50 genes including *GLDC,* which has been described previously (Table 1) [43]. Treatment was initiated on DOL 8 and MRS (repeated on DOL 18) showed reduced brain glycine levels (Table 5) which corresponded with clinical improvements in tone, encephalopathy, independent respiratory effort and feeding abilities. The infant developed focal and myoclonic seizures as well as burst suppression pattern on EEG. Levetiracetam was started with only a partial clinical response, thus a KD was initiated on DOL 30. CSF and brain glycine levels assessed on DOL 37 on KD showed equivalence to those obtained while on high dose benzoate therapy (Tables 4, 5). Due to difficult to manage perturbations in serum electrolyte levels and frequent emesis the KD was discontinued after 1 week and high dose sodium benzoate therapy was resumed.

Clinically, at age 13 months, the infant has made minimal developmental progress and is entirely gastrostomy tube fed, except for some tastes of purees for pleasure. She has periods of time when she is awake and alert, and able to focus her gaze on caregivers. She does not track and has presumed cortical visual impairment. She has axial hypotonia and has developed peripheral hypertonia. Though the infant has seizure-free days, on average, she continues to have tonic seizures that occur 1-2x/day and myoclonic seizures that cluster and can occur multiple times a day. She has significant neuro-irritability which has been challenging to manage.

### Case 3

Following an uncomplicated pregnancy and delivery, the infant presented on DOL 2 with progressive lethargy. Biochemical studies were consistent with NKH and molecular genetic testing revealed compound heterozygous mutations in *GLDC* (Table 1). MRS (obtained on DOL 52) showed a small glycine peak (Table 5).

The KD was initiated at 6 months of age due to intractable epilepsy with seizures occurring on average 4–5 times/day on clobazam therapy, but as frequent as 30 times/day. The patient remains on clobazam, but since KD initiation she has experienced over the course of one month a significant reduction in seizure frequency with seizures occurring on average 4 times/week which was maintained. She has remained on the KD for 9 months. At age 15 months, she can hold her head upright for a few seconds and can occasionally roll from back to front. She cannot roll from front to back and cannot sit independently. She can hold and track large objects like a cup, but she is not tracking smaller objects. She smiles occasionally and makes vocalizations but has no clear words yet.

### Case 4

The patient was born at term following an uncomplicated pregnancy and delivery. She had been briefly admitted during the first two weeks of life for feeding difficulties that self-resolved. She presented at 10 weeks of life for evaluation of abnormal eye movements, a dysconjugate gaze, and abnormal motor movements Biochemical studies were consistent with the diagnosis of NKH and molecular genetic testing showed compound heterozygous variants in *GLDC* (Table 1).

The patient’s seizures improved with optimization of sodium benzoate and dextromethorphan dosing and after 10 days the patient demonstrated increased alertness and eye contact. The infant was followed as an outpatient and started on clobazam for seizures. She made some developmental progress with attainment of intentional smile, increased head control and improvements in oral feeding skills. In the second year of life, the patient’s seizure frequency worsened, and she developed abnormal, ballistic-like, fidgeting movements and self-harming behaviors. Additional antiepileptics phenobarbital and topiramate were added and the patient’s sodium benzoate was progressively increased up to 600 mg/kg/day due to poorly controlled glycine levels. During this time, she became dependent on tube feedings.

Given these progressive difficulties, the ketogenic diet (KD) was initiated at 26 months of age. Since KD initiation, the patient has been able to achieve stable glycine levels with a reduction in sodium benzoate therapy and discontinuation of topiramate without a need for additional antiepileptics. She has remained on the ketogenic diet for over two years. Her background EEG and seizure control have improved. She remains on clobazam and a low dose of phenobarbital. The patient’s behaviors have improved, she is more relaxed and has fewer abnormal movements. She displays increased eye contact and social engagement with verbal skills limited to babbling. She has improved head control and axial tone and is able to crawl. Overall, the patient has tolerated the KD well. During vomiting illness, she requires gastrostomy feeds and her sodium benzoate dose is decreased to avoid low glycine levels. She remains on citrate therapy for hypercalciuria.

### Case 5

Following an uncomplicated pregnancy and delivery, the infant presented with seizures at 2 weeks of life. In retrospect, hypotonia had been noted at birth. On initial presentation, the infant experienced clusters of 4–5 seizures in a cluster that occurred on average 7–10 times/day. Biochemical testing and molecular genetic testing were consistent with the diagnosis of NKH. (Table 1). By 6 weeks, treatment with sodium benzoate and topiramate was initiated and the patient showed improvements in attention, appetite and seizure frequency.

The infant was started on KD at 9 weeks of life as an adjunct to pharmacologic therapy for epilepsy. No apparent clinical improvements were noticed following diet initiation. At his current age of 9 months, the infant has remained on the diet for approximately 7 months. He has clusters of 2–3 seizures/day and is managed on levetiracetam, clobazam and cannabidiol in addition to the KD. The KD is well tolerated and is taken by mouth in addition to solid foods. The infant remains globally severely delayed but is making slow progress with developmental therapies and can roll from stomach to back and push his head and upper body up when in a prone position. He has cortical visual impairment and nystagmus, but he is making progress in visual tracking.

### Case 6

The infant presented at 2 months of life in the setting of a viral illness with lethargy, decreased oral intake and abnormal movements. In retrospect, he was reported to have been unusually sleepy during the first weeks of life which was attributed to a difficult, vacuum-assisted, delivery. An EEG recording at age 2.5 months showed hypsarrhythmia, spasms and focal tonic seizures, and treatment was initiated with levetiracetam and vigabatrin. Subsequent biochemical evaluations were consistent with the diagnosis of NKH and confirmed by molecular studies (Table 1). Upon receipt of this diagnosis, treatment with vigabatrin was discontinued (due to its association with developmental regression in individuals with NKH [1, 44]) and clobazam was initiated, in addition to sodium benzoate therapy. The infant’s seizures initially improved and became less intense, but by age 5 months the seizures again increased in frequency and became associated with activity, prompting a dose escalation of all antiepileptic therapies.

The KD was initiated at 6 months of age. Since starting the KD, the infant’s seizures have decreased in frequency and severity. A continuous EEG obtained prior to diet initiation showed numerous clusters of infantile spasms lasting 7–35 min in duration and at least 3 focal seizures. Prior to KD, the patient’s parents reported average 4 seizures per day with average duration of 6 min. On KD, his parents report an average 1 seizure per day, with an average length of 2 min. The patient experienced 56 days with mild seizures and 7 seizure free days on his first 100 days on KD. Currently, he continues to have myoclonic seizures, and tonic seizures and spasms. A continuous EEG obtained while on the KD showed 1 cluster of spasms, 2 brief subclinical focal seizures and mild improvement in background activity. The infant is now 10 months old and has remained on the KD for over 4 months with good tolerance. He has made slow improvements in head control, and will roll with assistance. He holds objects and brings his hands to his mouth. He has a spontaneous smile and makes baby vocalizations. He has cortical vision impairment and hyperopic astigmatism but is able to intermittently fix and follow toys. He takes both formula and solid foods.

## Results

Patients’ clinical and molecular data are summarized in Table 1.

**Table 1:** Overview of patient molecular, biochemical and clinical data

Patient	1	2	3	4	5	6
Plasma glycine µmol/L (at diagnosis)	820	2108	1410	744	838	459
CSF glycine µmol/L (at diagnosis)	88	439	345	71	119	118.8
Ratio CSF:plasma glycine	0.11	0.21	0.24	0.10	0.14	0.26
Molecular diagnosis	*AMT*: c. c.959G > A p.(Arg320His)^a^ and c.515 T>C p.(Leu172Pro)	*GLDC:* c.1545G>C p.(Arg515Ser)^a^ and whole gene deletion (8.8 Mb 9p-deletion)^d^	*GLDC:* c.1166 C>T p.(Ala389Val)^b^ and c. 1401 + G>A	*GLDC:* c.806C>T, p.(Thr269Met)^b^ and c.1952A>G,p.(His651Arg)	*GLDC:* Homozygous^c^ c.2311G>A,.(Gly771Arg)	*AMT:* c.959C>G p.(Arg320His); c.1063del, p.(Ser355Leufs*2)
Age at presentation	DOL 3	< DOL 2	DOL 3	10 weeks	2 weeks	2 months
Predicted phenotype	Attenuated-poor	Severe	Attenuated-poor	Attenuated-Intermediate	Severe	Severe
Indication for KD initiation	Refractory epilepsy	Seizures, encephalopathy and poor prognosis	Refractory epilepsy	Escalating benzoate requirement, abnormal movements, self-harming behaviors and increasing antiepileptic medications	Seizure escalation	Adjunctive therapy
Age at diet initiation	6 months	1 month	6 months	26 months	9 weeks	6 months
SB dose prior to KD (mg/kg/d)	475	550	480	390 → 600	500	400
SB dose on KD (mg/kg/d)	200	325	128	300	290	200
Clinical impact	Increased alertness, decreased spasticity	Unable to assess d/t limited length of trial	Decreased seizure frequency (~ 4/day to ~ 4/week)	Decreased seizure frequency and antiepileptic medications, decreased hyperactivity, decreased self-harming behaviors	Difficult to evaluate d/t short time on benzoate therapy only	Decreased seizure intensity
KD ratio	3:1	3:1	3:1	3.2: 1	3.25:1 to 4:1	3:1
Tolerance	Discontinued after ~ 6 months Remains on SB 450 mg/kg/d	Discontinued at ~ 1 week Remains on SB 650 mg/kg/d	Remains on diet > 11 months Well-tolerated	Remains on diet > 2 years Well-tolerated	Remains on diet > 14 months Well-tolerated	Remains on diet > 6 months Well-tolerated

Summary of the pertinent biochemical, molecular, and clinical data available at the time of patients’ diagnosis used to predict the long-term phenotypes and summary of details regarding the indication for ketogenic diet initiation, procedure for initiation, and any observed benefits and side effects

Normal plasma glycine levels in neonates are 125–450 µM, normal CSF glycine levels in neonates are 4.7–15.4 µM, and the normal CSF:plasma glycine ratio is ≤ 0.02

CSF, cerebral spinal fluid; DOL, day of life; KD, ketogenic diet; SB, sodium benzoate, expressed in mg/kg/day; d/t, due to

^a^Known variant without residual activity upon expression and associated with severe outcome

^b^Known variant with residual activity upon protein expression in COS cells and associated with attenuated outcome

^c^Biallelic with exonic copy number variant excluded

^d^9p-deletion syndrome on one allele as previously published^44^

### Patient characteristics

The study included 6 patients with NKH recruited across 4 different institutions. The patients’ disease phenotypes were of varying severities—3 were predicted to have a severe phenotype, 2 attenuated-poor and 1 attenuated-intermediate. The patients presented between 2 days and 10 weeks of life and were diagnosed based on biochemical and molecular findings (Table 1). Four patients had pathogenic changes in the *GLCD* gene and 2 in the *AMT* gene. The KD was initiated between age 1 month and 26 months with indications for KD initiation including refractory epilepsy (4 patients), worsening clinical status and escalating benzoate requirements (1 patient) and as an adjunctive therapy (1 patient).

### Effect of KD on clinical status

#### Tolerance of KD

The diet was well-tolerated in all but one patient (patient 2) in whom the KD was discontinued after a 1-week trial due to electrolyte perturbations. Given the limited duration of this patient’s trial on the KD, observations regarding her clinical course are excluded from the summary of clinical results below. The KD was discontinued in patient 1 after approximately 14 months due to feeding intolerance at high levels of ketosis but that also has continued despite KD discontinuation, in addition the benefit of the diet not being impactful enough to outweigh the effort required to maintain it. The other patients have remained on the diet for a duration of 6 months to greater than 2 years (patient 4).

#### Impact on seizure control

Three participants demonstrated improved seizure control. Patient 3 initiated the KD at 6 months of age due to intractable epilepsy with seizures occurring on average 4–5 times/day to on average 4 times/week, which has been maintained over the 9 months of diet. Patient 4 experienced worsening seizure frequency and severity at the time of KD at 26 months. With KD initiation her background EEG improved and her seizure frequency decreased. Patient 6 initiated the KD at 6 months as an adjunctive therapy. Prior to starting the KD, a continuous 24-h EEG recording showed numerous clusters of infantile spasms lasting 7–35 min in duration and at least 3 focal seizures. His parents reported on average 4 seizures per day with an average duration of 6 min. On KD, his parents now report an average 1 seizure per day, with an average length of 2 min. The patient experienced 56 days with mild seizures and 7 seizure free days on his first 100 days on KD. A continuous 24-h EEG obtained while on the KD showed 1 cluster of spasms, 2 brief subclinical focal seizures and mild improvement in background activity. Patient 1 did not show a substantial improvement in seizure control on KD and required the addition of a third antiepileptic medication (lacosamide) in addition to continued topiramate and clobazam use in order to achieve adequate seizure control. Patient 5 was started on the KD at 6 months due to refractory epilepsy. He has remained on the diet for 7 months and experiences clusters of 2–3 seizures/day, an improvement from the 4–5 seizure/cluster that occurred on average 7–10 times/day at the time of diagnosis; however, firm conclusions regarding the impact of the KD on his seizure control cannot be drawn due to its close temporal proximity to starting other pharmacologic agents such as cannabidiol. The number of anticonvulsant drugs was unchanged in patients 2 and 5, decreased by 1 in patient 4, increased by 1 in patient 1 and delayed increased by 1 in patients 2 and 6.

### Impact on other neurologic characteristics

The clinical impact of the KD on other features such as exam findings and neurobehavioral status was variable. Patient 4 (with attenuated-intermediate NKH) showed the most favorable response. She started the KD for worsening behavioral issues including hyperactivity and self-harming behaviors, which have substantially improved. Patient 1 showed improvements in her level of alertness and head control, and increased grasping of objects, and on exam demonstrated improvement in spasticity with resolution of clonus. The remaining patients have not shown clear benefit. The KD has not had a significant impact on altering the neurodevelopmental course in any of the patients described.

### Effect of KD on plasma glycine levels

The levels of plasma glycine on high dose benzoate compared to ketogenic diet (KD) with low dose benzoate are displayed in Table 2. All 6 patients had significantly lower average plasma glycine levels on the KD (paired Wilcoxon rank sum test *p* = 0.028) with an average decrease of the mean plasma glycine level by 28%. Individually, this difference reached statistical significance in patients 1, 4 and 5 where sufficient data existed.

**Table 2:** Average plasma glycine levels comparing high dose benzoate to ketogenic diet with low dose benzoate

Patient	Glycine on benzate	N	Glycine on KD	N	Decrease	t-test p
Patient 1	307 ± 41.8	10	197 ± 74.8	10	36%	**0.001**
Patient 2	321.3 ± 91.5	8	248.3 ± 120	3	23%	0.41
Patient 3	299 ± 27	3	221.7 ± 91	3	26%	0.28
Patient 4	346.3 ± 80.2	34	222.4 ± 83.5	12	36%	**0.0003**
Patient 5	284.3 ± 80.1	4	191.3 ± 60.3	26	33%	**0.01**
Patient 6	184.2 ± 33.2	10	159.5 ± 44.1	13	13%	0.16

Comparison of the average glycine levels on high dose benzoate treatment compared to the glycine levels on ketogenic diet with low dose benzoate. N = total number of measurements. Glycine levels within the same patient are compared by a Student t-test, with p values < 0.05 considered significant in bold highlight. A paired Wilcoxon signed rank test comparing the average glycine levels between the two treatment modalities was significant at *p* = 0.028

Benz, benzoate

The glycine index is a personalized metric of an individual’s glycine metabolism, which is largely determined by the individual’s endogenous glycine synthesis and use, and residual glycine cleavage enzyme activity, but also takes into account dietary glycine intake and current benzoate dose (Fig. 1B) [20]. Without intervention this is a constant on a per weight basis over time [20]. Patients 1, 2 and 4, for which data were available, demonstrated a reduction in the glycine index on the KD to approximately 50% in patient 1, 25% in patient 2, and 50% in patient 4 (Table 3). This reflects an increase in the endogenous glycine use, compatible with the use of glycine for gluconeogenic purposes.

**Table 3:** The glycine index in nonketotic hyperglycinemia during ketogenic diet

Treatment	Weight (Kg)	Benzoate (mg/kg/day)	Glycine intake (mg/day)	Glycine index (mmol/kg/day)
*Patient 1*				
Benzoate	3.42	450	128	− 2.62
Benzoate	7.738	434	302	− 2.49
KD	8.705	230	210	− 1.27
*Patient 2*				
Benzoate	3.415	609	163	− 3.59
KD	4.17	264	273	− 0.96
Benzoate	4.25	602	178	− 3.62
*Patient 4*				
Benzoate	13.6	600	380	− 3.79
KD	17.8	300	35	− 2.06

Comparison of the glycine index on sodium benzoate therapy and on ketogenic diet

KD, ketogenic diet

### Effect of KD on central nervous system glycine levels

Serial measurements of CSF glycine levels comparing levels on the KD to levels at the time of diagnosis were obtained in patients 1 and 2 (Table 4). This showed that CSF glycine levels were lowered, by ~ 70% and ~ 85% respectively, but not normalized. CSF serine levels on the KD were also decreased to at or below the reference range, and CSF threonine levels were decreased to the lower end of the reference range. This is consistent with the use of these amino acids in states of glucose deprivation for gluconeogenic purposes (Fig. 1A).

**Table 4:** Cerebrospinal fluid levels of metabolites at the time of diagnosis and on ketogenic diet

CSF metabolite	Glycine (µM)			Serine (µM)		Threonine (µM)		Homocysteine (µM)	5-Methyl-tetra-hydrofolate (nm)
Time obtained	Diagnosis	KD	Off KD	Diagnosis	KD	Diagnosis	KD	KD	KD
*Patient 1*									
Values	88	23.7	40	54.3	32.9	71.7	16.8	8.9	123
Reference range	4.7–15.4	2.9–10.9	2.9–10.9	42.9–91.6	37.8–65.5	33.4–101.9	16.1–63.8	4.7–12.4	40–187
*Patient 2*									
Values	439	67		59.1	40	75.8	27.5	N/A	N/A
Reference range	4.7–15.4	4.7–15.4		42.9–91.6	42.9–91.6	33.4–101.9	33.4–101.9	N/A	N/A

Comparison of the levels of glycine and other relevant CSF metabolites utilized for gluconeogenic purposes pre- and post-implementation of the ketogenic diet

Age-appropriate reference range are provided for glycine, serine and threonine, which reflects the decrease observed in the first months of life. The neonatal values were developed from in 35 neonates in the first month of life [45]. Glycine, serine, threonine and homocysteine levels are in µmol/L and 5-methyltetrahydrofolate level in nmol/L

KD, ketogenic diet; N/A, not available

Serial brain MRS studies were obtained in patients 1 and 2 (Table 5). Prior to treatment, the value for brain glycine was increased 6.1–6.5 times above the average of control values in the basal ganglia. The glycine levels in the white matter were less than half those in the basal ganglia. Treatment with doses of benzoate sufficient to reduce plasma glycine levels in the therapeutic range was associated with a substantial reduction in glycine in the brain, reflected in both the absolute glycine value and in the glycine/creatine ratio. In the basal ganglia of patient 1 the glycine value decreased to 68% of pretreatment value and the glycine/creatine ratio decreased to 72% of pre-treatment value. In patient 2, the glycine/creatine ratio decreased to 68% of pre-treatment value. In the white matter for patient 1 the glycine value decreased to 55% of pretreatment value and the glycine/creatine peak height ratio decreased to 49% of pretreatment value. In patient 2, the glycine/creatine ratio decreased to 47% of pretreatment value. Despite these reductions, levels did not normalize. With KD the basal ganglia glycine values remained elevated to 4.2 times the average of controls for patient 1 and 6.8 times for patient 2. In patients 1 and 2, the glycine peak height ratios remained respectively elevated to 1.77 times and 1.90 times the average control values. Taking the margin of error of the assays into account, this reduction was equivalent to the reduction seen with high dose sodium benzoate therapy (Table 5). Brain choline levels in basal ganglia were in the low normal range in patient 1 and below normal in patient 2. These improved with benzoate and even more so with KD. Creatine and N-acetylaspartate levels were in the normal range. In patient 3, the glycine/creatine ratio decreased, but the impact of the mild age-related increase in creatine signal in infancy complicates interpretation [46, 47].

**Table 5:** Magnetic resonance spectroscopy data of the brain in nonketotic hyperglycinemia patients

Age	Treatment	Glycine plasma	Glycine	Creatine	PhCholine	NAA	mI + Gly	Gly/Crea	Gly + mI/crea	Gly/crea
Days			Value (SD%)	Value (SD%)	Value (SD%)	Value (SD%)	Value (SD%)		Height ratio 35 ms	Height ratio 144 ms
*Basal ganglia*										
Patient 1										
5	None	820	7.55 (9%)	7.87 (4%)	2.45 (5%)	5.42 (7%)	12.21 (14%)	0.96	0.86	0.76
17	Benzoate	147	5.17 (16%)	8.31 (5%)	2.99 (6%)	6.96 (7%)	12.23 (20%)	0.62	0.61	0.55
181	Benzoate	352	5.02 (12%)	12.04 (4%)	3.63 (5%)	11.72 (4%)	5.02 (12%)	0.42	0.34	0.27
230	KD + LDB	237	5.64 (12%)	11.48 (4%)	3.17 (5%)	11.32 (5%)	5.64 (12%)	0.48	0.39	0.33
413	KD + LDB	231	NA	NA	NA	NA	NA	0.25	0.36	0.27
Patient 2										
4	None	2108	NA	NA	NA	NA	NA	NA	0.87	0.98
18	Benzoate	403	8.04 (7%)	9.85 (4%)	1.72 (5%)	5.81 (6%)	8.04 (7%)	0.82	0.65	0.59
36	KD + LDB	263	8.23 (10%)	7.91 (6%)	2.78 (7%)	5.62 (9%)	9.60 (25%)	1.04	0.77	0.69
Patient 3										
52	Benzoate	281	NA	NA	NA	NA	NA	NA	0.21	NA
498	KD + LDB	190	NA	NA	NA	NA	NA	NA	0.16	NA
*Control neonates (N = 9)										
AVG range*	N/A		1.23 (0.0–3.10)	7.18 (3.77–11.4)	3.32 (2.25–4.42)	5.29 (3.46–7.83)	6.84 (4.31–9.81)	0.18 (0.0–0.31)	0.47 (0.36–0.59)	0.31 (0.21–0.38)
*White matter*										
Patient 1										
5	None	860	2.95 (18%)	3.22 (7%)	1.73 (5%)	2.54 (10%)	8.72 (17%)	0.91	NA	0.91
17	Benzoate	147	1.63 (64%)	5.00 (10%)	2.50 (8%)	4.60 (13%)	8.18 (35%)	0.33	NA	0.45
181	Benzoate	352	2.89 (22%)	7.46 (5%)	2.65 (5%	8.91 (4%)	4.49 (43%)	0.39	NA	0.28
230	KD + LDB	237	3.02 (15%)	6.84 (5%)	2.47 (5%)	8.46 (4%)	3.02 (15%)	0.44	NA	0.31
413	KD + LDB	231	NA	NA	NA	NA	NA	0.49	NA	0.37
Patient 2										
4	None	2108	NA	NA	NA	NA	NA	NA		1.26
18	Benzoate	403	1.14 (45%)	3.51 (7%)	1.72 (5%)	2.65 (9%)	8.12 (17%)	0.23	NA	0.59
36	KD + LDB	263	3.16 (17%)	3.10 (10%)	1.44 (8%)	2.96 (12%)	3.16 (17%)	1.02	NA	0.77

Magnetic resonance spectroscopy data of the brain obtained at obtained at TR 2000, TE 144 ms in patients with NKH with a voxel in the medial globus pallidus and in the frontal subcortical white matter. Values shown are the metabolite levels obtained from LCModel with the % standard deviation of the measurements. Control data on neonatal brain MRS glycine levels were extracted using identical processing from MRS data obtained over the basal ganglia of neonates imaged for possible hypoxic-ischemic injury, but who were later determined to have normal imaging and MRS findings. Therefore, only basal ganglia reference ranges are available

Gly, glycine; Crea, creatine; mI, myoinositol; PhCholine, choline and phosphocholine; NAA, N-acetylaspartate; KD, ketogenic diet; benzoate, high dose benzoate; LDB, low dose benzoate, for dosing see Table 1; SD, standard deviation; NA, not assessed

## Discussion

This study shows that the combination of ketogenic diet and low dose sodium benzoate therapy reduces plasma glycine levels and provides more stable low glycine levels than high dose benzoate therapy alone. A ketogenic ratio of 3:1 was enough to yield this marked glycine reducing effect even in the most severe patients. The effect occurred rapidly within 24 h concurrent with onset of ketosis, and thus a prompt adjustment of benzoate dosing was required. We recommend halving the benzoate dose at initiation of KD.

Despite apparent improvements in plasma biochemistry with glycine levels in the low normal range, the impact on the target organ, the brain, as measured via MRS was less robust. Independent of the method used, benzoate or KD, a reduction in plasma glycine corresponded with a reduction in brain glycine levels as assessed by MRS by approximately one third in the basal ganglia and by about half in the white matter (Table 5). Despite this significant reduction, levels still remained elevated above normal levels.

A reduction of CSF glycine has been reported in previous studies [29–34]. While strongly reduced, CSF glycine levels still remained elevated to above the normal range for patients 1, 2 and 4, who had serial CSF samples obtained on benzoate therapy, and for patients 1 and 2 on the KD. This differs from what was demonstrated in the NKH mouse studies, where brain glycine levels normalized with benzoate treatment [23].

The likely mechanism whereby KD causes this reduction in the glycine pool lies in the use of glycine for gluconeogenesis elicited by the strong carbohydrate restriction. Thus, as a glycine reduction strategy the target of the KD in NKH is not ketosis but rather glucose restriction, although the ketosis could have additional benefits as well. This increased endogenous usage of glycine for gluconeogenesis is reflected in a markedly reduced glycine index and associated reduction in the required dose of the benzoate (Table 2). This alters the equilibrium between the endogenous synthesis of glycine and catabolism via the glycine cleavage system in a substantive way (Fig. 1B). This gluconeogenetic use most likely involves a two-step process, first the conversion of glycine to serine by serine hydroxymethyltransferase, and then the use of serine dehydratase enzyme to provide pyruvate for gluconeogenesis (Fig. 1A). The decreased levels of CSF serine and threonine (Table 3) likely are reflective of their gluconeogenic metabolism to pyruvate through serine dehydratase and threonine dehydrogenase respectively. This exacerbates the already low serine levels in NKH, but the differential impact on the stereoisomers D-serine and L-serine has not been examined [45]. The transformation of glycine into serine involves serine hydroxymethyltranferase enzyme using 5,10-methylenetetrahydrofolate. It is unexplained why the CSF levels of 5-methyl-tetrahydrofolate and homocysteine measured while on KD in patient 1 remained normal (Table 3).

The critical enzyme in this mechanism, serine dehydratase, is highly expressed in the liver [48], but is not expressed in the brain [49]. Glycine-N-acyltransferase, which is critical in the mechanism of glycine conjugation to benzoate, is also expressed only in liver and secondarily in the kidney. Thus, the mechanism of glycine reduction in both benzoate therapy and KD involves enzymes that act through the liver, but not the brain. The expression of key enzymes in the liver but not the brain explains why both methods of glycine reduction were able to bring plasma glycine levels to the low normal range but did not fully correct brain glycine levels, and why these methods demonstrate equivalency in terms of brain glycine lowering capacity. We infer that it is thus unlikely that other measures that correct plasma glycine levels in the liver, such as liver-directed gene therapy or liver transplantation, will not fare any better at correcting brain glycine levels [23].

It is remains unclear which of the two methods of glycine reduction is clinically advantageous. The correction of plasma glycine levels on KD appeared to be more robust and stable than with benzoate; however, these data show equivalence in brain glycine lowering. Similar to what has been published about benzoate therapy [4, 21, 35–37], both methods appear to result in similar limited neurological clinical improvements in terms of increased alertness, easier epilepsy control compared to untreated, and some improvements in neurodevelopment. Patients 3, 4 and 6 showed objective improvements in seizure control with initiation of the KD, patient 4 exhibited significantly improved behavior and patient 1 displayed subjective improvements in her mental status and demonstrable improvement in her tone and degree of spasticity. The KD was trialed for just a 1-week period in patient 2, so it is difficult to draw conclusions on its clinical impact, whereas other patients remained on KD for at least 6 months with persistent beneficial impact. Given the limited duration of the study, the long-term impact of the combination of the KD and low dose sodium benzoate therapy on the clinical and biochemical stability remains to be documented.

**Fig. 2:**
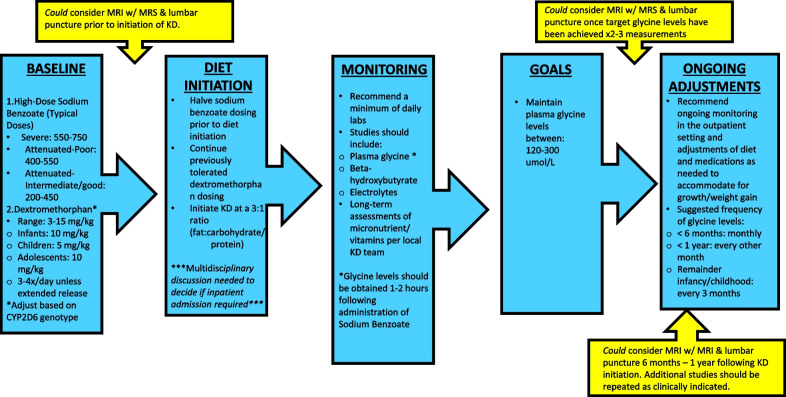
Proposed protocol for initiation of ketogenic diet in nonketotic hyperglycinemia

As for adverse effects, high dose benzoate therapy has a repugnant taste, carries a risk of gastric irritation, and of accidental overdosing with benzoate with potential life-threatening toxicity [20, 35, 36]. Benzoate acceptance is poor particularly in adult patients, and glycine control is often very variable, as reflected that even with good effort glycine levels in several patients on high dose benzoate were above the intended target range. The KD requires close subspecialty and dietary follow-up to avoid nutritional deficiencies and higher levels of ketosis may result in feeding intolerance as seen in patients 1 and 2. The KD also requires more effort and, in some cases, cost on the part of parents and caregivers to maintain when children are not formula fed, yet, half the families preferred KD over benzoate for the long term. Secondary biochemical effects of both therapeutic approaches should also be considered. As discussed above, there are indications that KD may exacerbate cerebral deficiency of serine and of 5,10-methylenetetrahydrofolate and the clinical impact of this is unknown. Presently, given our limited sample size and the observational nature of the study, our results do not clearly advocate for one method of glycine reduction over the other, and this will need to be addressed in future studies. Instead, clinicians in consultation with caregivers will need to evaluate the described benefits and risks and assess whether the KD be considered for each patient on a case-by-case basis. A guide to practical initiation KD in NKH based on our experience is provided in Fig. 2.

There are several limitations of this study resulting from its observational nature. Standardized tools for collection of clinical exam findings, developmental quotients and parent reports were not employed nor were EEGs obtained at standardized timepoints and recall bias on seizures numbers cannot be excluded. Only two patients had serial lumbar punctures and MRI and MRS studies. These factors limit our ability to draw definitive conclusions regarding the clinical impact of the KD. Ideally, prospective clinical controlled studies will be required to evaluate this. This study included mostly patients at the severe end of the phenotypic spectrum. Future studies should include more patients with attenuate-intermediate phenotypes (such as patient 4) and attenuated-good phenotypes, where KD may have greater clinical benefit. Given, its potential impact on neurocognitive symptoms [50], including mood, attention, and sleep quality, in addition to seizures, the effect of the KD on individuals with NKH who are higher-functioning is of interest. Such prospective study can also document appropriate benzoate dosing in this KD setting for patients of varying NKH severity.

Brain glycine measurement using MRS with LCModel analysis could be a potential new biomarker for the study of NKH and should be explored in a systematic study. Basal ganglia brain glycine levels were more elevated than white matter brain glycine levels at the time of diagnosis. This difference remained stable over the treatment course even over a 6-month period in patient 1. Regional differences should be explored in postmortem brain and using MRS technology in NKH patients, which may provide insight into both the pathogenesis of specific types of neurologic symptoms in NKH and what clinical parameters are likely to be most sensitive to therapeutic manipulations of brain glycine levels. Control data documenting normal glycine levels in different brain tissue types as measured via brain MRS will be valuable to properly compare the efficacy of different therapeutic interventions [51, 52].

In conclusion, the ketogenic diet with low dose benzoate therapy is an effective glycine reduction therapy for nonketotic hyperglycinemia. Compared to high dose benzoate, it was more effective at lowering plasma glycine levels. Both methods equally lowered brain glycine levels by about a third to half. Still, brain glycine levels remained elevated above levels seen in controls. Since both methods act through liver-mediated enzymatic reactions, complete correction of brain glycine accumulation will likely require a different mechanism that is more cerebral biochemistry based such as brain-based gene therapy or other brain-based biochemical manipulations. Brain glycine levels using MRS with LCModel analysis should be explored as a biomarker of the impact of new therapies in NKH.

## Data Availability

The data that supports the findings of this study are available in the manuscript.

## Abbreviations

CSF: Cerebrospinal fluid
DOL: Day of life
EEG: Electroencephalogram
IRB: Institutional review board
KD: Ketogenic diet
MRI: Magnetic resonance imaging
MRS: Magnetic resonance spectroscopy
NKH: Nonketotic hyperglycinemia
NMDA: *N*-methyl-d-aspartate
