# Correction: Ketogenic diet as a glycine lowering therapy in nonketotic hyperglycinemia and impact on brain glycine levels

**DOI:** 10.1186/s13023-023-02646-0

**Published:** 2023-03-13

**Authors:** Emily Shelkowitz, Russell P. Saneto, Walla Al-Hertani, Charlotte M. A. Lubout, Nicholas V. Stence, Mark S. Brown, Patrick Long, Diana Walleigh, Julie A. Nelson, Francisco E. Perez, Dennis W. W. Shaw, Emma J. Michl, Johan L. K. Van Hove

**Affiliations:** 1 Section of Clinical Genetics and Metabolism, Department of Pediatrics, University of Colorado, Education 2 South, L28-4114, East 17th Avenue, Aurora, CO 80045 USA; 2 Division of Pediatric Neurology, Department of Neurology, Center for Integrative Brain Research, Seattle Children’s Research Institute, Seattle Children’s Hospital, Seattle, WA 98105 USA; 3 Division of Genetics and Genomics, Boston Children’s Hospital, Harvard Medical School, Boston, MA USA; 4 Section of Metabolic Diseases, Beatrix Children’s Hospital, University of Groningen, University Medical Center, Groningen, Groningen, The Netherlands; 5 Department of Radiology, University of Colorado, Aurora, CO USA; 6 Section of Child Neurology, Department of Pediatrics, University of Colorado, Aurora, CO USA; 7 Department of Radiology, Seattle Children’s Hospital, University of Washington, Seattle, WA USA

**Correction : Orphanet Journal of Rare Diseases (2022) 17:423** 10.1186/s13023-022-02581-6

Following publication of the original article [1], we have been notified that reference 33 has error in the published year. The correct reference should be as follows:

33. Bzduch V, Behulova D, Kolnikova M, Payerova J, Fabriciova K. Ketogenic diet in nonketotic hyperglycinemia. J Inherited Metab Dis. 2010;33(Suppl 1):S31.
